# High-Dose Subcutaneous Immunoglobulins for the Treatment of Severe Treatment-Resistant Polymyositis

**DOI:** 10.1155/2014/458231

**Published:** 2014-07-16

**Authors:** Cherin Patrick, Delain Jean-Christophe, Crave Jean-Charles, Cartry Odile

**Affiliations:** ^1^Department of Internal Medicine, Pitié-Salpetrière Hospital Group, 47-83 Boulevard de l'Hôpital, 75013 Paris, France; ^2^Octapharma, 92100 Boulogne-Billancourt, France; ^3^Clinique Mutualiste Catalane, 66000 Perpignan, France

## Abstract

Polymyositis is a rare debilitating condition characterized by chronic inflammation and muscle weakness. Standard treatments include corticosteroids and immunosuppressants; however, resistance to these regimens may develop. Intravenous immunoglobulins (IVIg) are thus recommended for patients with drug-resistant polymyositis. The patient presented a resistant polymyositis with severe muscle weakness, increasing dysphagia, and significant loss in weight. Subcutaneous immunoglobulins (SCIg) were initiated after failure of steroids and immunosuppressive drugs. SCIg was given twice per week (2 then 1.3 g/kg/month). Clinical recovery was observed within 2 months after the SCIg initiation. After several injections, the patient showed a progressive improvement in muscle strength. Serum creatine kinase activity decreased to normal levels, and dysphagia was resolved. The SC injections were generally well tolerated and good patient satisfaction was reported. This promising observation suggests that SCIg may be useful in active and refractory polymyositis.

## 1. Introduction

Polymyositis is a chronic inflammatory disorder affecting mainly the proximal skeletal muscles. This disease is associated with increased mortality and morbidity, particularly relating to life-threatening muscle weakness and visceral involvement [1–3]. Due to its low prevalence of approximately 6-7 cases per 100 000 subjects, few randomized trials have been conducted in polymyositis to define the optimal therapy [4].

To date, standard treatments include corticosteroid therapy, as a first-choice treatment, and then immunosuppressive therapy in the case of steroid-related side effects or inefficacy [5]. Intravenous immunoglobulin (IVIg) therapy is recommended in patients with polymyositis refractory to corticosteroids and immunosuppressive agents, despite the lack of randomized controlled studies [6–8]. Given the intravenous route of administration and related hospitalizations, this therapy shows an economic burden and a significant impact on patient's quality of life. Therefore, subcutaneous self-administered injections were developed as an alternative therapy to intravenous injections, but granted indications are still limited.

We report here a case of steroid/immunosuppressant resistant polymyositis, with esophageal involvement, that was successfully treated with subcutaneous immunoglobulins (SCIg).

## 2. Case Presentation

A Caucasian woman was referred to us with 6-year history of polymyositis, started at 43 years old. She presented severe proximal muscular weakness in the upper and lower limbs without involvement of wrist or finger flexors and increasing difficulty with standing. She had also developed dysphagia, which consequently caused weight loss of 4 kg during the last 6 months. Laboratory results revealed elevated serum creatine kinase (CK) activity (397 IU/L, normal <211 UI/L).

Polymyositis was diagnosed in 2006 while she was pregnant. The diagnosis of polymyositis was confirmed by a muscle biopsy, according to the International Consensus Criteria [9]. Muscle biopsy showed endomysial inflammatory infiltrates (CD8 T-Cells) surrounding and invading the nonnecrotic muscle fibers and a ubiquitous expression of MHC-1 by the noninvaded muscle cells. Rimmed vacuoles, ragged red fibers, and cytochrome oxidase-negative fibers suggesting inclusion body myositis were not observed.

According to the international criteria for polymyositis, the patterns of weakness were bilateral, symmetrical, and only proximal, with involvement of neck flexors. Electromyography showed increased insertional and spontaneous activity in the form of fibrillation potentials, positive sharp waves, and the presence of short duration, small amplitude, and polyphasic motor unit action potentials (MUAPs). Myositis-specific antibodies were negative. Muscle MRI was not performed as it was considered not useful for the diagnosis [9].

Intravenous immunoglobulin (IVIg, 2 g/kg/month) associated with bolus corticosteroids was therefore initiated; a significant improvement was then noticed. This treatment was followed by a maintenance therapy, during 2007, including corticosteroids and immunosuppressive treatment with either methotrexate or azathioprine, without IVIg. In September 2008, the patient showed a severe relapse despite treatments; consequently she received a course of rituximab which consisted of 4 infusions (375 mg/m^2^ each) given weekly. In January 2009, immunosuppression with cyclosporine was started. Due to worsening of clinical results, plasma exchange (16 courses) was introduced in June 2010, in association with IVIg (2 g/kg/month), corticosteroids (20 mg daily), and immunosuppressive therapy with tacrolimus (4 mg, twice daily). Six months later, plasma exchange and IVIg were discontinued due to catheter-related bacteremia, and lower doses of tacrolimus were consequently given for one month (3 mg, twice daily). Meanwhile, she was admitted to an intensive outpatient physiotherapy program (4 sessions per week). In February 2011, corticosteroid treatment was reduced to 10 mg daily and tacrolimus was unchanged (4 mg, twice daily). In September 2012, she experienced worsening dysphagia with weight loss (4 kg between December and June 2012, from 48 to 44 kg). Plasma exchange was then reintroduced (12 courses), combined with IVIg (2 g/kg/month).

The patient was referred to us in November 2012. Plasma exchange and tacrolimus were discontinued and anti-interleukin-1 (anti-IL-1) was introduced to existing treatments including IVIg (2 g/kg/month) and corticosteroids (10 mg daily). Anti-IL-1 showed no clinical benefit. Due to difficult venous access, frequent hospitalizations, and the clinical benefit of immunoglobulins, SCIg (Gammanorm, 60 mL twice per week or 2 g/kg/month) was initiated in February 2013 after discussion with the patient. All other medications were stopped. At that time, the patient had severe muscle weakness; she was unable to walk or stand unaided. She was experiencing severe dysphagia which led to further loss of weight (4 kg, from 44 to 40 kg). Muscle weakness score was 55/88 (normal strength: 88 points) [6] and myositis activity scale was 49/75 (maximum disability: 75 points) [7]. CK activity was 397 UI/L. The patient was motivated for this subcutaneous treatment that was expected to prevent hospital readmissions and potential complications related to the intravenous therapy, such as her previous catheter-related bacteremia. Before the initiation of SCIg, a second muscle biopsy confirmed the active polymyositis.

**Figure 1:**
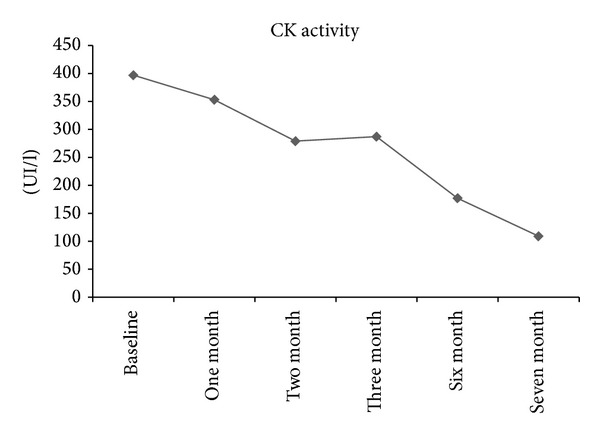
Changes in creatine kinase (CK) activity over time after SCIg initiation. Improvement in CK values was observed from the first month after the initiation of SCIg, up to 7 months.

**Figure 2:**
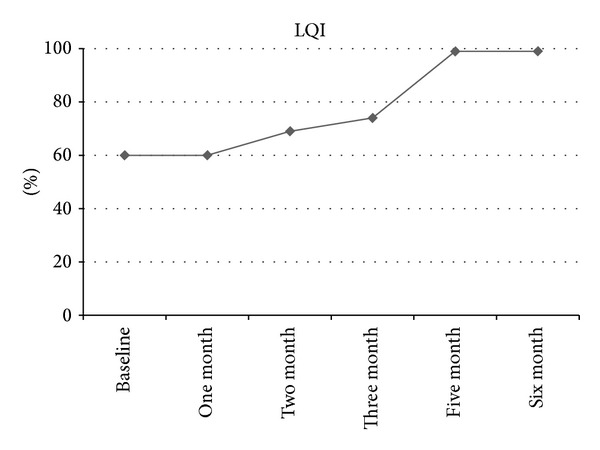
Changes in the Life Quality Index (LQI) over time after IgSC initiation. The Life Quality Index (LQI) was used to assess the treatment satisfaction. This scale comprises 18 items ranging from 1 to 7. The sum of scores is then adjusted to obtain a total score of 100 points. Improvement in LQI was observed starting from two months after the initiation of SCIg and reaching the maximum values after five months.

Shortly after the first SCIg injection, the patient experienced a headache. This event was considered as related to the treatment. Therefore, SCIg was reduced to 40 mL (1.3 g/kg/month) twice per week. Two months later, her CK activity was decreased to 279 UI/L but yet no benefit was observed on the clinical status. After several courses, she showed an increasing improvement in clinical and biological parameters (Figure 1). Meanwhile, she stayed one month in an inpatient physiotherapy rehabilitation department, followed by an outpatient physical therapy program. In September 2013, CK activity was normal (177 UI/L). She was able to walk unaided and dysphagia was resolved. Neurological examination showed only mild weakness in the lower limbs. Muscle weakness score (72/88) and myositis activity scale (26/75) had improved markedly. The Life Quality Index (LQI) reflected high quality of life due to immunoglobulin treatment; score was 99% (Figure 2). No side effects were reported; particularly no local pain or rash was observed. The patient was satisfied with the SCIg injections since it was administered at home. She also reported satisfaction in achieving clinical improvement and in enjoying meals again. She showed an increase in her body weight (from 40 Kg to 45 Kg). To meet the patient's demand, the SCIg treatment was reduced to one injection (40 mL) per week. However, one month later the CK activity increased to 285 UI/L and then to 417 UI/L a couple of weeks later, with slight physical relapses. Subsequently, the previous regimen was resumed.

## 3. Discussion

This case shows that SCIg was safe and effective in a patient with polymyositis, presenting increasing dysphagia and subsequent weight loss, despite several lines of treatment. The patient age at disease onset, the pattern of weakness, and the two muscle biopsies confirm the definite polymyositis diagnostic.

Significant improvement in muscle strength was observed after several courses of SCIg (1.3 g/kg/month; 40 mL twice weekly). The treatment with SCIg successfully treated dysphagia and reduced physical disability, thus preventing parenteral nutrition and allowing for normal daily activities. A slight relapse was observed when the SCIg was reduced to 0.7 g/kg/month (or 40 mL once weekly). This observation suggests that continuous higher dosage of SCIg may be advisable particularly in this case.

The clinical benefit of IVIg was recently reported in autoimmune-mediated disorders affecting nerves and muscles, including multifocal motor neuropathy, Guillain-Barré syndrome, and chronic inflammatory demyelinating polyneuropathy [10]. The subcutaneous administration of Ig has been initiated in these diseases [11–14]; few studies have reported the safety and efficacy of SCIg [15, 16].

In polymyositis and dermatomyositis, Danieli et al. described the benefit of SCIg in 7 patients with resistant disease [17]. SCIg was administered at usual IVIg monthly dose, fractioned into equal doses given weekly (in average 0.2 g/kg/week). After a median follow-up of 14 months, patients showed a favorable clinical response and improved quality of life [17]. SCIg was well tolerated in patients with PM/DM disease; no particular safety concerns were raised [17, 18].

The SCIg treatment did not result in severe adverse events. Our patient experienced a headache only after the first injection. This adverse event was considered in relation to the treatment and resolved with dosage reduction (from 2 g/kg/month, 60 mL twice weekly to 1.3 g/kg/month, 40 mL twice weekly). In general, local adverse events such as redness and swelling at the injection site are frequent with SCIg treatment [15, 16, 19]. Given the SC route of administration and the reduced doses given on a weekly schedule, systemic reactions and thromboembolic events seem to be less frequent in SCIg, compared with IVIg [20]. According to the few published reports, SCIg injections were well tolerated in patients PM/DM [17, 18]; no major adverse events were reported. No patients reported severe, local, or systemic reactions. Mild local reactions including swelling, redness, and burning sensation were reported in few patients at the infusion site that disappeared within 2 days [17, 18]. No other adverse events were reported with SCIg therapy.

Further advantages of SCIg include the home-based setting and the ease of handling infusions, which prevent hospitalizations, reduce costs, and contribute to patient autonomy [21]. Switching from IVIg to SCIg was associated with the increased quality of life and significant improvement in treatment satisfaction [22]. Consistently, our patient reported higher satisfaction and less emotional distress with the subcutaneous self-administered treatment.

In conclusion, the SCIg dose should be tapered to achieve long-lasting clinical benefit whilst preventing adverse events. This promising observation suggests that SCIg may be useful in active and refractory polymyositis, although further investigations are required to confirm these findings.
