# Corrigendum to “High-Dose Subcutaneous Immunoglobulins for the Treatment of Severe Treatment-Resistant Polymyositis”

**DOI:** 10.1155/2015/436736

**Published:** 2015-04-09

**Authors:** Patrick Cherin, Jean-Christophe Delain, Jean-Charles Crave, Odile Cartry

**Affiliations:** ^1^Department of Internal Medicine, Pitié-Salpetrière Hospital Group, 47-83 Boulevard de l'Hôpital, 75013 Paris, France; ^2^Octapharma, 92100 Boulogne-Billancourt, France; ^3^Clinique Mutualiste Catalane, 66000 Perpignan, France

In the paper titled “High-Dose Subcutaneous Immunoglobulins for the Treatment of Severe Treatment-Resistant Polymyositis,” the authors were cited as “Cherin Patrick, Delain Jean-Christophe, Crave Jean-Charles, and Cartry Odile.” This is now replaced by the correct names as above.

